# A Retrospective Analysis of the Robson Classification Implementation in a Tertiary Care Hospital in Serbia

**DOI:** 10.3390/jcm14082700

**Published:** 2025-04-15

**Authors:** Svetlana Jankovic, Marija Rovcanin, Ana Tomic, Aleksa Jokic, Konstantin Kostic, Tijana Grujic, Natasa Karadzov Orlic

**Affiliations:** 1 Clinic for Gynecology and Obstetrics, Narodni Front, Kraljice Natalije 62, 11000 Belgrade, Serbia; svetlanajankovic.r@gmail.com (S.J.);; 2 Faculty of Medicine, University of Belgrade, Dr Subotica Starijeg 8, 11000 Belgrade, Serbia; 3 Center for Radiology and Magnetic Resonance Imaging, University Clinical Center of Serbia, Pasterova 2, 11000 Belgrade, Serbia

**Keywords:** cesarean section, Robson classification, delivery, rate

## Abstract

**Background/Objectives**: Cesarean section (CS) is an essential intervention in obstetric care, significantly contributing to reducing the rate of maternal and neonatal mortality and morbidity. It has been recommended that the acceptable CS rate should not go beyond 10–15% across all deliveries. Nonetheless, the CS rate has escalated over the past decades. To understand the factors contributing to the rise in CS rates, the Robson classification that relies on pre-labor, intrapartum, and postpartum parameters has been proposed. As no journal-reported data are currently available on the implementation of the Robson classification in Serbia, we aimed to identify trends in CS rates, as well as the Robson groups with the highest risk for CS at our tertiary care clinic. **Methods**: We conducted a retrospective, cross-sectional analysis of 6574 women who gave birth to live fetuses weighing a minimum of 500 g and with a gestational age of at least 22 weeks. **Results**: The overall CS rate was 30.5%, with a statistically significant difference in CS rates between different Robson groups (*X*^2^ = 2703.9, *p* < 0.001). Robson groups 1 (31.9%), 3 (30.4%), and 5 (10.3%) were the largest, and groups 9 (0.9%) and 7 (1.3%) were the smallest. The CS rate in group 5 was the highest (30.3%), followed by groups 1 (20.3%) and 2 (13.2%). Group 5 was the largest contributor to the absolute CS rate (9.25%), followed by groups 1 (6.21%) and 2 (4.03%). **Conclusions**: We effectively implemented Robson classification for monitoring CS rates and distinguishing specific groups that individually contribute to these rates.

## 1. Introduction

Cesarean delivery (CD) is a regularly performed surgical procedure and an essential measure in obstetric care, substantially aiding in the reduction in maternal and newborn mortality and morbidity rates [1]. The World Health Organization (WHO) has recommended that the acceptable CD rate should not go beyond 10–15% across all deliveries [2]. Nonetheless, the CD rate has risen throughout the 21st century, revealing variability among various healthcare institutions, both within countries themselves and across nations [3,4], accompanied by an increasing occurrence of CD deliveries lacking substantial medical rationale [5]. The aforementioned increase in CD rates has underscored its impact on various aspects of obstetric care, mainly on maternal and neonatal health [3,6]. This tendency results in a significant surplus in healthcare expenditures that could be redirected towards more pressing health issues [7], simultaneously exacerbating interclass disparities in healthcare access [8]. The excessive utilization of medical interventions, including CD, may lead to a series of auxiliary procedures that could potentially be circumvented [9]. It is crucial to conduct CDs when it is medically warranted in order to safeguard the health and welfare of both mothers and neonates in current and subsequent pregnancies [10].

To understand the factors contributing to the rise in CD rates and to establish appropriate methods and/or tools for assessment, it was indispensable to create an international classification system for CDs. The proposed Robson classification relies on pre-labor, intrapartum, and postpartum parameters regularly obtained in maternity units globally [11]. This classification of CD is widely endorsed by the WHO [12,13]. The classification framework enables users to more effectively assess and comprehend delivery circumstances, clinical customs, indications, and outcomes, with an outlook on critical epidemiological aspects. The indications would enhance the evaluation and analysis of factors affecting the CD rate, allowing for comparisons among institutions on both national and global scales [14]. Moreover, the United Nations emphasizes its value as a guiding and benchmarking system, particularly in environments where access to highly qualified personnel or advanced diagnostic equipment is limited [15]. The strength of the Robson system lies in its simplicity and reliance on basic, routinely collected obstetric data [16]. This makes it accessible and applicable across a wide range of healthcare settings, allowing for consistent monitoring, comparison, and improvement in cesarean section practices [17].

The efficacy of the Robson categorization system has been recognized over the past decade, with a growing number of facilities and countries implementing it to assess and analyze CD rates [18]. The Robson classification is extensively used across the globe in hospital-based and nationwide studies, mainly with examples from Europe, including the Nordic countries (Denmark, Finland, Iceland, Norway, and Sweden [19]), Greece [20], Switzerland [21], and Spain [22], and based on the data from the Euro-Peristat study reported by Zeitlin et al. [14], many other European countries such as France, Belgium, the Netherlands, Germany, Italy, Slovenia, Latvia, Estonia, Malta, and Cyprus. This similarly applies to numerous countries in Asia, Africa, North America, and South America (Argentina, Brazil, Cambodia, China, Democratic Republic of the Congo, Ecuador, India, Japan, Kenya, Mexico, Nepal, Nicaragua, Niger, Nigeria, Paraguay, Peru, Philippines, Sri Lanka, Thailand, Uganda, and Vietnam [4]), including the United States of America (USA) [23] and Canada [24].

The fundamental objective of the Robson Classification system is to establish a standardized and consistent approach for large-scale evaluation, monitoring, and comparing cesarean delivery rates within and between national [25] and worldwide [4] healthcare contexts throughout time. This method categorizes all deliveries into ten distinct, mutually exclusive groups based on six essential obstetric criteria [11] (labor onset, parity, gestational age, fetal presentation, number of fetuses, and the data about previous CD [11,26]), enabling us to ascertain which groups most significantly influence the overall and increasing cesarean delivery rates. Ultimately, comprehensive evaluations of the Robson method furnish evidence-based justification for policy formulation and the creation of measures designed to optimize CD utilization and enhance mother and newborn outcomes [27]. These insights facilitate the assessment of clinical care protocol implementation. The systematic application of the Robson classification in healthcare institutions will aid in identifying the precise types of pregnancies that adversely affect the CD rate of the institution, applicable in both single-center and broader contexts, facilitating policies that could result in a decrease in inadequately indicated CDs [28,29,30]. The regular reporting and application of this classification system have proven to be highly effective. In a teaching hospital in the Netherlands, the daily documentation of Robson classification groups, along with a detailed analysis of CD indications within each group, led to a reduction in unnecessary CDs over the course of a one-year follow-up period [31]. Furthermore, targeted interventions can be implemented to modify CD rates within specific Robson groups. An Australian study employed the Robson categorization to identify methods for decreasing cesarean delivery rates in specific populations. The authors recommended a greater degree of initiative in providing external cephalic conversion to all women with breech presentation and suggested evaluating vaginal breech delivery under explicitly specified criteria for appropriate candidates [32]. Furthermore, they underscored the necessity of vigilant oversight of cohorts undergoing labor induction, aiming toward lowering the incidence of inductions conducted prior to 41 weeks of gestation [32]. Moreover, in further detailed analysis, Robson groups can be compared based on other patient characteristics (obesity, comorbidities, etc.) and whether other confounding factors could influence the risk for certain Robson groups [33].

Based on a comprehensive literature search, no journal-reported data are currently available on the implementation of the Robson classification in Serbia, as this study appears to be the first analysis of this kind in a tertiary healthcare institution in Serbia. Therefore, the aim of this study was to utilize the Robson classification system to identify patterns associated with specific Robson groups at our tertiary care clinic while examining the Robson groups that significantly contribute to the overall CD delivery rates.

## 2. Materials and Methods

### 2.1. Study Design

This study was a retrospective, cross-sectional analysis conducted at our clinic in Belgrade. This establishment is a tertiary care hospital that manages all categories of pregnancies, including high-risk cases, from different regions across Serbia. The eligible participants comprised women who delivered within the one-year interval from 1 January 2023 to 31 December 2023.

### 2.2. Participants

This study’s population comprised 6574 consecutive women who gave birth throughout the study period, specifically focusing on those who underwent CD with live fetuses weighing a minimum of 500 g and with a gestational age of at least 22 weeks. Fetuses classified as stillborn were omitted from this study to concentrate the analysis exclusively on live births. The exclusion was implemented to maintain consistency in the assessment of CD rates and the distribution of Robson groups, as the inclusion of stillborn cases could introduce confounding variables that might influence the interpretation of data and designated outcomes.

### 2.3. Data Collecting Tools

The data for this study were obtained from the hospital’s clinical information system, which encompasses all protocols and patient records of the women and their newborns. The Robson classification was established based on six predefined obstetric characteristics of pregnant women: parity (nulliparous and multiparous women), history of previous cesarean section, mode of birth onset (spontaneous, induced, or pre-labor CD), number of fetuses (singleton or multiple), gestational age (preterm or term), and fetal presentation (cephalic, breech, or transverse) [11,13]. We categorized the pregnancies referred for delivery into one of ten groups based on observed features, as presented in Table 1.

### 2.4. Ethical Consideration

This study was implemented in accordance with the International Code of Medical Ethics of the World Medical Association (Declaration of Helsinki) and approved by the institution’s Ethical Committee (decision number 22008 2023 020849; date of approval: 23 October 2023).

### 2.5. Statistical Analysis

Out of descriptive statistics methods, frequencies and percentages (%) were computed for all noted Robson groups and subdivisions. The CD rate for the year 2023 at our clinic, the comparative size of each Robson group, and the CD frequencies for specific Robson groups are shown as numbers and percentages. Absolute CD rates for each Robson group in relation to total births as well as their concordant relative CD rates concerning the total number of CDs were computed and shown as percentages and rankings. The chi-square test was employed to assess the statistical significance of the variations in the number of CDs among the various groups. To assess the risk of cesarean section in specific Robson groups, an odds ratio (OR) with a 95% confidence interval was computed. The significance threshold was established at *p* < 0.05. Statistical analyses were conducted with SPSS software version 29.0 (IBM Corp. Released 2020. IBM SPSS Statistics for Windows, Version 29.0. Armonk, NY, USA: IBM Corp.)

## 3. Results

The data were collected from 6574 medical files of women and their deliveries. The overall CD rate in the population was 30.5%.

As presented in Table 2, Robson groups 1 (31.9%), 3 (30.4%), and 5 (10.3%) were the largest groups, and groups 9 (0.9%) and 7 (1.3%) were the smallest. The CD rate in group 5 (prior cesarean) was the highest (30.3%), followed by groups 1 (20.3%) and 2 (13.2%).

As presented in Table 3, group 5 was the largest contributing group to the absolute CD rate (9.25%), followed by groups 1 (6.21%) and 2 (4.03%). Conversely, groups 3, 4, 7, and 9 made minimal contributions to the total CD rates (each exhibiting an absolute group contribution to overall CD rate below 1%). Relative contribution to the overall CD rate presented with equivocal results and rankings.

**Figure 1:**
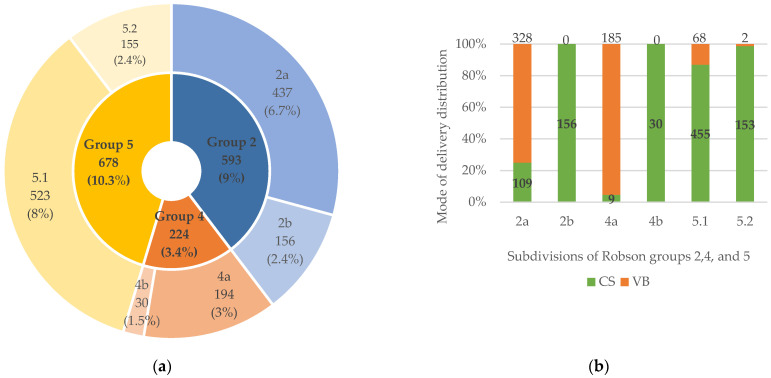
Robson groups 2, 4, and 5 and their subdivisions’ (2a, 2b, 4a, 4b, 5.1, and 5.2) distributions and rates, with a presentation of the subdivisions of cesarean delivery (CD) distributions: (**a**) a nested chart presenting subdivisions’ distribution across their respective Robson groups (percentages (%) were calculated based on total sample numbers); (**b**) a bar chart presenting the number of CDs (lower, bolded numbers) and vaginal births (VBs, upper non-bolded numbers) across each analyzed subdivision, with a graphical representation of CD rates (% on the left axis) within each subdivision group.

Subdivisions of Robson groups 2, 4, and 5 are shown in Figure 1. Within class 2, subgroup 2a (induced labor) included 437 women (6.7% of all classes), of whom 328 underwent CD, accounting for 5.4% of the total CD rate. Subgroup 2b (CD performed before labor) consisted of 156 women, contributing to an overall rate of 7.8%.

In group 4, subgroup 4a (induced labor) comprised 194 women, with a low CD rate of 0.5%, while subgroup 4b (all of whom underwent CD before labor) included 30 women, representing a rate of 1.5%.

For group 5, subgroup 5.1 (only one previous CD) included 523 cases (8% overall), with 455 women undergoing CD, resulting in a high CD rate of 22.7%. In subgroup 5.2 (women with at least two previous CDs), 155 women had a CD, with a rate of 7.6%.

The chi-squared test showed statistically significant differences in CD frequency between different Robson groups (*X*^2^ = 2703.9, *p* < 0.001), which was further explored using regression analysis. As all women in group 9 were indicated for CD, they were excluded from the regression analysis.

Utilizing logistic regression analysis (Table 4), the differences in mode of birth (specifically the odds ratio (OR) for CD delivery) were assessed among groups with varying modes of labor onset. Specifically, group 1 (nulliparous, singleton term pregnancies) and group 3 (multiparous, singleton term pregnancies without prior CD) served as reference groups (spontaneous labor) and were compared to groups with induced labor (groups 2a and 4a). The induction of labor elevates the chances of CD delivery (OR = 1.791; 95% CI 1.434–2.236, *p* < 0.001). The risk was identified in nulliparous women (OR = 1.374; 95% CI 1.079–1.751; *p* = 0.010), but not in the multiparous cohort (OR = 1.628; 95% CI 0.794–3.339; *p* = 0.183).

## 4. Discussion

Our study showed that during a one-year period, our tertiary care clinic had 6574 births, with an overall CD rate of 30.5%. Robson groups 1, 3, and 5 were the largest groups, as well as group 9 (0.9%). The cesarean rate in group 5 (prior cesarean) was the highest, followed by groups 1 and 2, them being the largest contributing groups to the absolute cesarean rate. Labor induction elevates the likelihood of necessitating a CD in comparison to the spontaneous labor onset. The potential risk was observed in nulliparous individuals, but not in multiparous groups.

The overall cesarean rate in our study was 30.5%, twice as high as the WHO-recommended rate. Based on the data from 2015, cesarean birth rates in European countries varied from 16.1% to 56.9% (with omitted data from Serbia) [14]. Comparable to our group, several countries exhibited analogous percentages, including Austria (29.2%), Ireland (31.3%), Germany (32.2%), and Portugal (32.9%) [14]. A 2019 single-center study conducted in Greece indicated that 60.9% of the sample underwent cesarean sections; however, as it was conducted in a private healthcare facility, this figure may not accurately represent the rates of cesarean sections at public health institutions [20]. Since the onset of the 21st century, there has been a documented rise in overall cesarean rates. Countries are exhibiting varying rates of increase, such as Denmark (16.4% to 20.7%), Norway (14.4% to 16.5%), and Sweden (15.5% to 17.1%) [19]. However, it is frequently noted that the rise in cesarean section rates can primarily be attributed to time-based increases [19], with the most significant absolute contribution from Robson group 5 (women with previous cesareans) [4,10,14,19,21], as well as group 2 (induction or pre-labor cesareans) [4,10,19], and group 1 (spontaneous term births) [4,21,34]. All identified groups exhibited the highest absolute and relative contributions to CD rates in our study cohort, as previously shown, in alignment with the patient demographics of a tertiary care clinic.

Vogel et al. [4] published findings that highlight the correlation between Robson group sizes, their CD rates, and their corresponding absolute contribution to overall CD rates, which are influenced by the Human Development Index (HDI) of the countries. In 2022, Serbia recorded a notably high HD of 0.805 [35] and displayed characteristic sizes and distributions of Robson groups, along with their cesarean section rates and contributions. In nations with a high HDI, it is widely perceived that groups 1 and 3 are analogous and the most populous; however, the contribution rates to civil society are predominantly shouldered by group 5, with a lesser extent by groups 1 and 2 [4]. This study used data exclusively from a tertiary care center. Consequently, the previously described outcomes may vary at the national level.

It is significant that in each Robson group, the cesarean section rates transcend the recommendations set forth by the WHO Robson guidelines [13]. The sole exceptions are group 3, with rates below the required 3% (2.9%), and group 9, where both the recommendation and practice are at a rate of 100%, consistent with our cohort. Likewise, additional studies revealed a 100% rate of cesarean section in group 9 [36,37,38]. This is significant as it reflects the quality of the collected data and proper classification.

Despite deviating from the indicated rates, the comparatively greater sizes of groups 8 (multiple pregnancies) and 10 (preterm, singleton, cephalic deliveries) align with anticipated outcomes in a tertiary healthcare center. The cesarean section rate in Robson group 1 (nulliparous women with singleton pregnancies in spontaneous labor) was 20.1%, significantly exceeding Robson benchmarks, which indicate that rates below 10% are optimal. The reported results may indicate a tendency to conduct unnecessary cesarean sections, mostly due to a low threshold for interpreting the criteria for such procedures. This method coincides with international trends advocating for a differentiated, tailored treatment strategy that evaluates the benefits and risks for both mother and child, offers comprehensive counseling, and respects the patient’s preferences [39]. Cephalopelvic disproportion may obstruct vaginal delivery [40,41]. Prolonged labor may result in maternal exhaustion, requiring a cesarean section for secure delivery. Indicators of impaired fetal health, including atypical heart rate patterns, may necessitate a cesarean section for prompt delivery [42]. Labor may be impeded by labor dystocia, characterized by insufficient cervical dilatation or inefficient uterine contractions [43]. All identified disorders are not uncommon in tertiary care settings, thereby elucidating the elevated CD rates in these populations.

This study’s population reveals that the amalgamation of ‘groups 3 and 4’ (33.0%) is inferior to that of ‘groups 1 and 2’ (40.0%), suggesting a marginally bigger nulliparous demographic. This may be typical in a tertiary care institution. Research indicates that nulliparous women devoid of risk factors experience significantly elevated rates of complex births compared to multiparous women without a prior cesarean section, even when the latter present several risk factors [44]. Women experiencing an extended latent phase were more prone to have obstetric procedures. Assisted vaginal delivery was more prevalent among nulliparous women experiencing a protracted latent phase, while emergency cesarean sections happened frequently more in both nulliparous and multiparous women with a longer latent phase [45]. Outlined the types of pregnancies that would be monitored and managed at a high-level care hospital, as is the practice at our institution.

It has been proposed that group 5 (multiparous with previous CD, single, cephalic pregnancies) should represent approximately fifty percent of the overall CD rate [13]. In this study, group 5 represented 30.3%, closely aligning with the overall CD rate of 30.5%. The size of group 5 exceeds the recommended CD rates. This may also result from a policy of scheduling pre-labor cesarean sections for all women with one prior cesarean, without attempting a trial of labor. In numerous obstetric units, the prevailing protocol is to evaluate the possibility of performing vaginal birth after cesarean section (VBAC) for women with a single prior cesarean section who possess no contraindications, while advising elective repeat CD for those with multiple prior cesarean sections or contraindications to VBAC, as this approach may decrease the CD rate in this group [46].

This study is, to our knowledge, the inaugural report of the analysis of cesarean sections utilizing the Robson classification in a tertiary healthcare institution in Serbia. While our data are derived from a singular tertiary care center with a considerable volume of referred cases, the findings may not accurately reflect national patterns and should not be generalized to the broader population. Thus, real-world data from a wider population across several centers may produce divergent results. This study presents the outcomes from a one-year period. A multi-year review of changes in the Robson categorization system would yield a more thorough understanding of its application, clinical efficacy, and prospective benefits across various healthcare environments. It should be noted that in contemporary obstetric practice, the Robson classification may primarily function as an indicative rather than a predictive instrument. Although it offers a uniform framework for categorizing and comparing cesarean birth rates across various groups and settings, its effectiveness in predicting the necessity or consequences of CDs is constrained. This brings the need to emphasize the necessity of incorporating categorization with additional clinical judgment and patient-specific variables when determining the manner of delivery.

## 5. Conclusions

Our study revealed that Robson groups 1, 3, and 5 were the primary contributors to the total number of deliveries performed. The overall CD rate at our clinic was 30.5%, with groups 5, 1, and 2 being the key contributors to this elevated rate. Furthermore, the induction of labor was identified as a significant factor increasing the likelihood of a CD delivery in comparison to spontaneous labor. This increased risk was observed in both primiparous and multiparous women, highlighting the impact of labor induction on delivery outcomes across different patient groups. This study aided in identifying effective interventions for monitoring CD rates and distinguishing specific groups that individually contribute to these rates at our tertiary care clinic. Future research should investigate targeted strategies to enhance obstetric practices, minimize needless CSs, and improve decision-making in obstetric care.

## Figures and Tables

**Table 1:** The Robson classification (modified from [13]).

Class	Description
1	Nulliparous, single cephalic, ≥37 weeks, in spontaneous labor
2	Nulliparous, single cephalic, ≥37 weeks, induced labor or CD before labor
2a	Induced labor
2b	CD before labor
3	Multiparous without previous CD, single, cephalic, ≥37 weeks, spontaneous labor
4	Multiparous without previous CD, single, cephalic, ≥37 weeks, induced labor or CD before labor
4a	Induced labor
4b	CD before labor
5	Multiparous with previous CD, single, cephalic, ≥37 weeks
5.1	With one previous CD
5.2	With two or more previous CDs
6	All nulliparous breeches
7	All multiparous breeches (including previous CD)
8	All multiple pregnancies (including previous CD)
9	All transverse or oblique lies (including previous CD)
10	All preterm single cephalic, <37 weeks (including previous CD)

CD—cesarean delivery.

**Table 2:** Distribution of this study’s population according to the Robson classification system.

Robson Group	Number of Women in Each Group	Robson Class Rates (%)	Number of CDs in Each Group	Overall CD Rates (%)	Group-Wise CD Rates (%)
1	2095	31.9%	408	20.3%	19.5%
2	593	9.0%	265	13.2%	44.7%
3	1999	30.4%	58	2.9%	2.9%
4	224	3.4%	39	2.0%	17.4%
5	678	10.3%	609	30.3%	89.7%
6	124	1.9%	105	5.2%	83.9.%
7	84	1.3%	50	2.5%	59.5%
8	289	4.4%	239	11.1%	76.8%
9	61	0.9%	61	3.0%	100.00%
10	427	6.5%	197	9.5%	44.5%
Total	6574	100.0%	2005	100.0%	

CD—cesarean delivery.

**Table 3:** Robson groups’ contribution to the CD rate.

Robson Group	Absolute Group Contribution to Overall CD Rate (%)	Rank for Absolute Contribution of Groups to CD Rates	Relative Group Contribution to Overall CD Rate (%)	Rank for Relative Contribution of Groups to CD Rates
1	6.21	2	20.35	2
2	4.03	3	13.22	3
3	0.88	8	2.89	8
4	0.59	10	1.95	10
5	9.25	1	30.32	1
6	1.58	6	5.19	6
7	0.76	9	2.49	9
8	3.38	4	11.07	4
9	0.93	7	3.04	7
10	2.89	5	9.48	5
Total	30.5		100.0	

CD—cesarean delivery.

**Table 4:** Logistic regression analysis.

Robson Groups	OR (CI)	*p* Value
Groups 2a and 4a vs. 1 and 3 (spontaneous vs. induced labor)	1.791 (1.434–2.236)	*p* < 0.001
Group 2a vs. 1 (nulliparous groups)	1.374 (1.079–1.751)	*p* = 0.010
Group 4a vs. 3 (multiparous groups)	1.628 (0.794–3.339)	*p* = 0.183

OR—odds ratio; CI—confidence interval.

## Data Availability

The data that support the findings of this study are available from the corresponding author (N.K.O.) upon reasonable request.

## Abbreviations

The following abbreviations are used in this manuscript:

CD	cesarean delivery
WHO	World Health Organization
USA	United States of America
OR	odds ratio
CI	confidence interval
HDI	Human Development Index

## References

[1] Betran A.P., Torloni M.R., Zhang J.J., Gülmezoglu A.M., WHO Working Group on Caesarean Section (2016). WHO Statement on Caesarean Section Rates. BJOG Int. J. Obstet. Gynaecol..

[2] WHO Statement on Caesarean Section Rates. https://www.who.int/news-room/questions-and-answers/item/who-statement-on-caesarean-section-rates-frequently-asked-questions.

[3] Angolile C.M., Max B.L., Mushemba J., Mashauri H.L. (2023). Global Increased Cesarean Section Rates and Public Health Implications: A Call to Action. Health Sci. Rep..

[4] Vogel J.P., Betrán A.P., Vindevoghel N., Souza J.P., Torloni M.R., Zhang J., Tunçalp Ö., Mori R., Morisaki N., Ortiz-Panozo E. (2015). Use of the Robson Classification to Assess Caesarean Section Trends in 21 Countries: A Secondary Analysis of Two WHO Multicountry Surveys. Lancet Glob. Health.

[5] Elnakib S., Abdel-Tawab N., Orbay D., Hassanein N. (2019). Medical and Non-Medical Reasons for Cesarean Section Delivery in Egypt: A Hospital-Based Retrospective Study. BMC Pregnancy Childbirth.

[6] Rahman M., Khan N., Rahman A., Alam M., Khan A. (2022). Long-Term Effects of Caesarean Delivery on Health and Behavioural Outcomes of the Mother and Child in Bangladesh. J. Health Popul. Nutr..

[7] Wu M.L., Nichols P.M., Cormick G., Betran A.P., Gibbons L., Belizan J.M. (2023). Global Inequities in Cesarean Section Deliveries and Required Resources Persist. Eur. J. Obstet. Gynecol. Reprod. Biol..

[8] Kundu S., Sharif A.B., Chowdhury S.S.A., Afroz S., Dey R., Hossain A. (2024). Socioeconomic and Geographical Inequalities in Delivery by Cesarean Section among Women in Bangladesh, 2004–2017. BMC Pregnancy Childbirth.

[9] Souza J., Gülmezoglu A., Lumbiganon P., Laopaiboon M., Carroli G., Fawole B., Ruyan P., The WHO Global Survey on Maternal and Perinatal Health Research Group (2010). Caesarean Section without Medical Indications Is Associated with an Increased Risk of Adverse Short-Term Maternal Outcomes: The 2004–2008 WHO Global Survey on Maternal and Perinatal Health. BMC Med..

[10] Tura A.K., Pijpers O., De Man M., Cleveringa M., Koopmans I., Gure T., Stekelenburg J. (2018). Analysis of Caesarean Sections Using Robson 10-Group Classification System in a University Hospital in Eastern Ethiopia: A Cross-Sectional Study. BMJ Open.

[11] Robson M. (2001). Classification of Caesarean Sections. Fetal Matern. Med. Rev..

[12] Opiyo N., Torloni M.R., Robson M., Ladfors L., Gholbzouri K., Kacerauskiene J., Vila-Candel R., Kessler J., Lucovnik M., Betrán A.P. (2022). WHO’s Robson Platform for Data-Sharing on Caesarean Section Rates. Bull. World Health Organ..

[13] Robson Classification: Implementation Manual. https://www.who.int/publications/i/item/9789241513197.

[14] Zeitlin J., Durox M., Macfarlane A., Alexander S., Heller G., Loghi M., Nijhuis J., Sól Ólafsdóttir H., Mierzejewska E., Gissler M. (2021). Using Robson’s Ten-Group Classification System for Comparing Caesarean Section Rates in Europe: An Analysis of Routine Data from the Euro-Peristat Study. BJOG Int. J. Obstet. Gynaecol..

[15] Robson Classification: Implementation Manual|United Nations in Azerbaijan. https://azerbaijan.un.org/en/51083-robson-classification-implementation-manual.

[16] Betrán A.P., Vindevoghel N., Souza J.P., Gülmezoglu A.M., Torloni M.R. (2014). A Systematic Review of the Robson Classification for Caesarean Section: What Works, Doesn’t Work and How to Improve It. PLoS ONE.

[17] Torloni M.R., Betran A.P., Souza J.P., Widmer M., Allen T., Gulmezoglu M., Merialdi M. (2011). Classifications for Cesarean Section: A Systematic Review. PLoS ONE.

[18] Betran A.P., Ye J., Moller A.-B., Souza J.P., Zhang J. (2021). Trends and Projections of Caesarean Section Rates: Global and Regional Estimates. BMJ Glob. Health.

[19] Pyykönen A., Gissler M., Løkkegaard E., Bergholt T., Rasmussen S.C., Smárason A., Bjarnadóttir R.I., Másdóttir B.B., Källén K., Klungsoyr K. (2017). Cesarean Section Trends in the Nordic Countries—A Comparative Analysis with the Robson Classification. Acta Obstet. Gynecol. Scand..

[20] Giaxi P., Gourounti K., Vivilaki V., Zdanis P., Galanos A., Antsaklis A., Lykeridou A. (2023). Implementation of the Robson Classification in Greece: A Retrospective Cross-Sectional Study. Healthcare.

[21] Triep K., Torbica N., Raio L., Surbek D., Endrich O. (2020). The Robson Classification for Caesarean Section-A Proposed Method Based on Routinely Collected Health Data. PLoS ONE.

[22] Gutiérrez-Martínez S., Fernández-Martínez M.N., Adánez-García J.M., Fernández-Fernández C., Pérez-Prieto B., García-Gallego A., Gómez-Salgado J., Medina-Díaz M., Fernández-García D. (2023). Applying the Modified Ten-Group Robson Classification in a Spanish Tertiary Hospital. J. Clin. Med..

[23] Smith D.C., Phillippi J.C., Lowe N.K., Breman R.B., Carlson N.S., Neal J.L., Gutierrez E., Tilden E.L. (2020). Using the Robson 10-Group Classification System to Compare Cesarean Birth Utilization Between US Centers With and Without Midwives. J. Midwifery Womens Health.

[24] Gu J., Karmakar-Hore S., Hogan M.-E., Azzam H.M., Barrett J.F.R., Brown A., Cook J.L., Jain V., Melamed N., Smith G.N. (2020). Examining Cesarean Section Rates in Canada Using the Modified Robson Classification. J. Obstet. Gynaecol. Can..

[25] Rocha A.S., Paixao E.S., Alves F.J.O., Falcão I.R., Silva N.J., Teixeira C.S.S., Ortelan N., Fiaccone R.L., Rodrigues L.C., Ichihara M.Y. (2023). Cesarean Sections and Early-Term Births According to Robson Classification: A Population-Based Study with More than 17 Million Births in Brazil. BMC Pregnancy Childbirth.

[26] Parveen R., Khakwani M., Naz A., Bhatti R. (2021). Analysis of Cesarean Sections Using Robson’s Ten Group Classification System. Pak. J. Med. Sci..

[27] Silva C.H.M., Laranjeira C.L.S. (2018). Use of the Robson Classification System for the Improvement and Adequacy of the Ways of Delivery in Maternities and Hospitals. An Opportunity to Reduce Unnecessary Cesarean Rates. Rev. Bras. Ginecol. E Obs. RBGO Gynecol. Obstet..

[28] Begum T., Rahman A., Nababan H., Hoque D.M.E., Khan A.F., Ali T., Anwar I. (2017). Indications and Determinants of Caesarean Section Delivery: Evidence from a Population-Based Study in Matlab, Bangladesh. PLoS ONE.

[29] Robson M., Murphy M., Byrne F. (2015). Quality Assurance: The 10-Group Classification System (Robson Classification), Induction of Labor, and Cesarean Delivery. Int. J. Gynecol. Obstet..

[30] Kacerauskiene J., Bartuseviciene E., Railaite D.R., Minkauskiene M., Bartusevicius A., Kliucinskas M., Simoliuniene R., Nadisauskiene R.J. (2017). Implementation of the Robson Classification in Clinical Practice:Lithuania’s Experience. BMC Pregnancy Childbirth.

[31] van Dillen J., Lim F., van Rijssel E. (2008). Introducing Caesarean Section Audit in a Regional Teaching Hospital in The Netherlands. Eur. J. Obstet. Gynecol. Reprod. Biol..

[32] Tanaka K., Mahomed K. (2017). The Ten-Group Robson Classification: A Single Centre Approach Identifying Strategies to Optimise Caesarean Section Rates. Obstet. Gynecol. Int..

[33] Crequit S., Korb D., Morin C., Schmitz T., Sibony O. (2020). Use of the Robson Classification to Understand the Increased Risk of Cesarean Section in Case of Maternal Obesity. BMC Pregnancy Childbirth.

[34] Einarsdóttir K., Sigurðardóttir H., Ingibjörg Bjarnadóttir R., Steingrímsdóttir Þ., Smárason A.K. (2019). The Robson 10-Group Classification in Iceland: Obstetric Interventions and Outcomes. Birth Berkeley Calif.

[35] United Nations (2024). Human Development Report 2023–24.

[36] Begum T., Nababan H., Rahman A., Islam M.R., Adams A., Anwar I. (2019). Monitoring Caesarean Births Using the Robson Ten Group Classification System: A Cross-Sectional Survey of Private for-Profit Facilities in Urban Bangladesh. PLoS ONE.

[37] Geze S., Tura A.K., Fage S.G., Van Den Akker T. (2021). Can the Robson 10 Group Classification System Help Identify Which Groups of Women Are Driving the High Caesarean Section Rate in Major Private Hospitals in Eastern Ethiopia? A Cross-Sectional Study. BMJ Open.

[38] Bolognani C.V., Reis L.B.D.S.M., Dias A., Calderon I.D.M.P. (2018). Robson 10-Groups Classification System to Access C-Section in Two Public Hospitals of the Federal District/Brazil. PLoS ONE.

[39] Reif P., Brezinka C., Fischer T., Husslein P., Lang U., Ramoni A., Zeisler H., Klaritsch P. (2016). Labour and Childbirth After Previous Caesarean Section: Recommendations of the Austrian Society of Obstetrics and Gynaecology (OEGGG). Geburtshilfe Frauenheilkd..

[40] Adeyanju B.T., Aduloju O.P., Okunola T.O., Ojo I.O. (2023). Head Circumference, as Predictor of Cephalopelvic Disproportion: A Prospective Analysis of Cases of Spontaneous Vaginal Delivery and Caesarean Section in Ekiti State, Nigeria. Afr. J. Reprod. Health.

[41] Pavličev M., Romero R., Mitteroecker P. (2020). Evolution of the Human Pelvis and Obstructed Labor: New Explanations of an Old Obstetrical Dilemma. Am. J. Obstet. Gynecol..

[42] Litorp H., Gurung R., Målqvist M., Kc A. (2020). Disclosing Suboptimal Indications for Emergency Caesarean Sections Due to Fetal Distress and Prolonged Labor: A Multicenter Cross-Sectional Study at 12 Public Hospitals in Nepal. Reprod. Health.

[43] Place K., Kruit H., Tekay A., Heinonen S., Rahkonen L. (2019). Success of Trial of Labor in Women with a History of Previous Cesarean Section for Failed Labor Induction or Labor Dystocia: A Retrospective Cohort Study. BMC Pregnancy Childbirth.

[44] Jardine J., Blotkamp A., Gurol-Urganci I., Knight H., Harris T., Hawdon J., van der Meulen J., Walker K., Pasupathy D. (2020). Risk of Complicated Birth at Term in Nulliparous and Multiparous Women Using Routinely Collected Maternity Data in England: Cohort Study. BMJ.

[45] Ängeby K., Wilde-Larsson B., Hildingsson I., Sandin-Bojö A.-K. (2018). Prevalence of Prolonged Latent Phase and Labor Outcomes: Review of Birth Records in a Swedish Population. J. Midwifery Womens Health.

[46] Strambi N., Sorbi F., Bartolini G.M., Forconi C., Sisti G., Seravalli V., Di Tommaso M. (2020). Non-Clinical Variables Influencing Cesarean Section Rate According to Robson Classification. Med. Kaunas Lith..

